# Scaling up Evidence-Based Interventions in US Public Systems to Prevent Behavioral Health Problems: Challenges and Opportunities

**DOI:** 10.1007/s11121-019-01048-8

**Published:** 2019-08-24

**Authors:** Abigail A. Fagan, Brian K. Bumbarger, Richard P. Barth, Catherine P. Bradshaw, Brittany Rhoades Cooper, Lauren H. Supplee, Deborah Klein Walker

**Affiliations:** 1 Department of Sociology, Criminology & Law, University of Florida, 3362 Turlington Hall, P.O. Box 117330, Gainesville, FL 32611-7330 USA; 2 Colorado State University, Fort Collins, CO USA; 3 School of Social Work, University of Maryland, Baltimore, Baltimore, MD USA; 4 Curry School of Education, University of Virginia, Charlottesville, VA USA; 5 Department of Human Development, Washington State University, Pullman, WA USA; 6 Child Trends, Bethesda, MD USA; 7 School of Public Health, Boston University, Boston, MA USA

**Keywords:** Scaling up, Type 2 research, Dissemination, Implementation, Evidence-based programs, Evidence-based policies, Behavioral health problems

## Abstract

A number of programs, policies, and practices have been tested using rigorous scientific methods and shown to prevent behavioral health problems (Catalano et al., Lancet 379:1653–1664, 2012; National Research Council and Institute of Medicine, 2009). Yet these evidence-based interventions (EBIs) are not widely used in public systems, and they have limited reach (Glasgow et al., American Journal of Public Health 102:1274–1281, 2012; National Research Council and Institute of Medicine 2009; Prinz and Sanders, Clinical Psychology Review 27:739–749, 2007). To address this challenge and improve public health and well-being at a population level, the Society for Prevention Research (SPR) formed the Mapping Advances in Prevention Science (MAPS) IV Translation Research Task Force, which considered ways to scale up EBIs in five public systems: behavioral health, child welfare, education, juvenile justice, and public health. After reviewing other efforts to scale up EBIs in public systems, a common set of factors were identified as affecting scale-up in all five systems. The most important factor was the degree to which these systems enacted public policies (i.e., statutes, regulations, and guidance) requiring or recommending EBIs and provided public funds for EBIs. Across systems, other facilitators of scale-up were creating EBIs that are ready for scale-up, public awareness of and support for EBIs, community engagement and capacity to implement EBIs, leadership support for EBIs, a skilled workforce capable of delivering EBIs, and data monitoring and evaluation capacity. It was concluded that the following actions are needed to significantly increase EBI scale-up in public systems: (1) provide more public policies and funding to support the creation, testing, and scaling up of EBIs; (2) develop and evaluate specific frameworks that address systems level barriers impeding EBI scale-up; and (3) promote public support for EBIs, community capacity to implement EBIs at scale, and partnerships between community stakeholders, policy makers, practitioners, and scientists within and across systems.

Prevention science is dedicated to promoting public health and well-being and preventing behavioral health problems^1^ at a population level (National Research Council and Institute of Medicine 2009). Behavioral health problems are of concern because they affect a large proportion of the population and contribute substantially to morbidity and mortality (Hale and Viner 2012; Patel 2012). They also have significant human and financial costs to public systems and taxpayers. One report estimated that mental, emotional, and behavioral health disorders cost the US 247 billion dollars in 2007 (National Research Council and Institute of Medicine 2009), but costs are likely even higher, given that 428 billion dollars are spent annually in the child welfare system alone to substantiate cases of child maltreatment (Peterson et al. 2018). Although some public health problems, such as tobacco use, have declined over the last decade, the prevalence of other types of drug use (e.g., opioids), depression, comorbid mental illness, and obesity have all increased (Center for Behavioral Health Statistics and Quality 2017; Hales et al. 2017). Moreover, improvements seen in the overall population are not enjoyed equally by population subgroups, and many behavioral health problems are more pronounced for racial and ethnic minority populations (Center for Behavioral Health Statistics and Quality 2017; Hales et al. 2017). Population health setbacks have occurred despite growing knowledge and an expanding evidence base about how to prevent these problems (Dodge 2018). Without concerted efforts to capitalize on scientific breakthroughs that address behavioral health problems *at scale*, the proportion of the population suffering from these problems and racial/ethnic health disparities is likely to worsen (Koh et al. 2010).

Decades of prevention science research indicate that behavioral health problems are both *developmental* in nature and *preventable* (Catalano et al. 2012; Coie et al. 1993). That is, these problems often have a predictable trajectory, with an onset early in the life course and peaks in prevalence rates thereafter (Halfon et al. 2014; Ward et al. 2017). Their developmental nature suggests that many disorders can be prevented, ideally by targeting the risk and protective factors experienced in multiple ecological contexts prior to the onset of problems (Coie et al. 1993; Kellam et al. 1999). Over the past few decades, rigorous evaluation studies have demonstrated that a number of programs, policies, and practices (i.e., evidence-based interventions [EBIs]) prevent the occurrence or reduce the prevalence of behavioral health problems and promote well-being by changing the prevalence and influence of risk and protective factors (Catalano et al. 2012; Hawkins et al. 2015; National Research Council and Institute of Medicine 2009).

There are indicators that scaling up EBIs can reduce behavioral health problems at a population level and result in significant financial savings for public systems. For example, after Washington State made significant financial investments in EBIs and implemented strategies such as early childhood education and family therapy for juvenile offenders across the state, Washington had lower rates of juvenile and adult crime compared to other states (Drake et al. 2009; Washington State Institute for Public Policy 2011). Similarly, Pennsylvania has implemented over 200 replications of a diverse menu of EBIs and seen significant reductions in juvenile justice and child welfare system utilization and lower prevalence rates of delinquency and out-of-home placement (Bumbarger 2013; Jones et al. 2008; Moore and Bumbarger 2011). The state of Maryland scaled up an EBI (Positive Behavioral Interventions and Supports, PBIS) in over 850 schools, and a quasi-experimental evaluation demonstrated significant impacts on a range of academic, behavioral, and discipline outcomes for students in the state (Pas et al. 2019). Randomized trials of the Communities That Care (CTC) and PROmoting School-community-university Partnerships to Enhance Resilience (PROSPER) prevention systems, which are two frameworks that support the scale-up of EBIs in communities, have demonstrated significant reductions in adolescent substance use and delinquency and improvements in academic achievement at population (i.e., city, county, and/or state) levels (Feinberg et al. 2007; Feinberg et al. 2010; Hawkins et al. 2009; Hawkins et al. 2012; Spoth et al. 2011; Spoth et al. 2015). Likewise, the scale-up of public health policies (such as increasing taxes on smoking and alcohol, creating smoke-free workplaces, and mandating motorcycle helmet usage) has been linked to population-level reductions in injury, illness, and deaths, as well as substantial financial savings (Community Preventive Services Task Force 2013; Trust for America's Health 2019; U.S. Department of Health and Human Services, Office of the Surgeon General 2016).

These examples provide evidence that it is possible to scale up EBIs and improve population health. Nonetheless, scaling up EBIs to have maximum impact at the population level remains one of the most vexing challenges facing prevention science (Catalano et al. 2012; Hawkins et al. 2015; Zerhouni 2003). Despite increased attention to the existence, importance, and cost-benefits of EBIs, EBIs represent only a small fraction of the programs delivered and policies enacted by public systems, and they reach a relatively small proportion of the individuals and communities that could benefit from them (Dodge 2018; Glasgow et al. 2012; National Research Council and Institute of Medicine 2009; Prinz and Sanders 2007). Many interventions implemented in public systems are reactive and focus on *treating* current problems rather than *preventing* disorders (U.S. Department of Health and Human Services, Office of the Surgeon General 2016). Although the USA spends about three trillion dollars per year on medical care, it spends less than 5% of this amount on preventive services (Trust for America's Health 2019; Walker 2016). An analysis of NIH funding of research grants and cooperative agreements in FY 2012–2017 indicated that only 17% of the projects and 23% of the funding were for prevention-related research (Murray et al. 2018).

The primary aim of this paper is to recommend ways to further advance the scaling up of EBIs to improve public health and well-being at a population level. Scaling up EBIs is a major goal of the Society for Prevention Research (SPR; (https://www.preventionresearch.org), whose mission is “*dedicated to advancing scientific investigation on the etiology and prevention of social, physical and mental health, and academic problems***and on the translation of that information to promote health and well-being** [emphasis added].” The current paper addresses this goal by leveraging the efforts and findings of the SPR Mapping Advances in Prevention Science (MAPS) II Translational Research Task Force. Operating from 2008 to 2014, the MAPS II Task Force provided a research agenda for prevention scientists to address the barriers that impede the adoption, implementation, and sustainability of EBIs across populations and settings (Spoth et al. 2013; Spoth et al. 2008). The MAPS II Task Force identified two main challenges facing prevention scientists: (1) determining ways to build local infrastructure to support EBIs and (2) identifying salient research questions and improving scientific methods to answer them.

In 2016, SPR formed the MAPS IV Translation Research Task Force to build on and extend the MAPS II efforts. This paper summarizes the MAPS IV Task Force findings regarding factors influencing and facilitating the uptake of EBIs after they have demonstrated effectiveness in rigorous evaluations. Whereas the MAPS II Task Force considered how prevention researchers could advance EBI implementation and dissemination, the MAPS IV Task Force was more outward-focused and examined how EBIs could be scaled up within *public systems*, specifically the behavioral health, child welfare, education, juvenile justice, and public health systems.^2^ The next sections of this paper explain our rationale for focusing on public systems, discuss the constraints and supports for EBI scale-up that exist across these systems, and provide recommendations for how to increase and evaluate EBI scale-up efforts.

## The Promise of Public Systems for Scaling up EBIs

The Society for Prevention Research charged the MAPS IV Task Force with identifying ways to increase the use of EBIs to improve health and well-being in communities, states, and the nation. Although more comprehensive definitions can be found elsewhere (e.g., Brown et al. 2017), we follow Spoth et al. (2013) and Gottfredson et al. (2015) in defining EBIs as those programs, policies, and practices that have been shown to improve health and well-being and reduce behavioral health problems when evaluated using rigorous scientific methods such as those outlined in the SPR Standards of Evidence (Gottfredson et al. 2015). We recognize that more research is needed to expand the number of available EBIs, especially rigorous studies comparing EBIs to interventions that are widely used in public systems (i.e., “treatment as usual”), and to better understand how EBI implementation quality and outcomes may vary across populations and contexts. We further acknowledge that scaling up EBIs is not the only method for improving health and well-being at a population level. Alternative strategies include, for example, promoting the scale-up of the prevention principles and practices that are most commonly used in systems (Green et al. 2009), and increasing the delivery of simple, inexpensive strategies (i.e., kernels) that may draw on elements common to EBIs but are easier to deliver and can reach more individuals (Embry 2004; Rotheram-Borus et al. 2012). Our charge was to consider how to scale up *existing EBIs* because a significant amount of human and financial resources has been spent developing and testing EBIs, yet these interventions are currently not being used at a scale sufficient to impact public health and well-being. As a result, society has not yet optimized its return on investment in developing and demonstrating the efficacy of these approaches.

Consistent with other definitions of “scale-up” (e.g., Gottfredson et al. 2015; Indig et al. 2018), we call for increased efforts to provide access to high-quality, sustained delivery of EBIs *at the level necessary to produce sustained, population-wide improvements in public health and well-being*. Access to EBIs is particularly important for disadvantaged populations (as defined by race/ethnicity, gender, disability, socioeconomic status, etc.) given the known disparities in behavioral health problems (National Academies of Sciences, Engineering, and Medicine 2017). Achieving health equity and promoting social justice are concurrent goals of SPR, and the SPR Disparities-Equity Task Force has been charged with considering ways to increase the effectiveness, transdisciplinary innovation, and cultural relevance of preventive interventions that enhance the quality of life for individuals and families from health disparity populations. In a forthcoming paper, the Task Force will summarize (a) definitional issues, (b) mechanisms and theoretical models, (c) preventive intervention designs, (d) methods, and (e) efficacious preventive interventions designed to reduce health disparities.

In the current paper, the MAPS IV Task Force took a public systems approach to scaling up EBIs because EBIs must be widely used to improve the health and well-being of the population and to reduce health disparities nationwide, and these goals are best achieved via public systems (Brown and Beardslee 2016). As shown in Table 1, public systems are charged and funded by the government to improve the behavioral health problems that EBIs target. Their funding, regulatory, and policy parameters both drive and constrain the use of EBIs. One or more federal agencies usually oversee the systems’ functions and activities, but individual states or counties also have the authority to set policies and distribute funds, and many prevention services are implemented at the local level. For example, the Substance Abuse and Mental Health Services Administration (SAMHSA), which oversees the behavioral health system, has provided Strategic Prevention Framework State/Tribal Incentive Gants (SPF SIG/TIGs) to support EBIs to prevent substance use to all 50 states, eight jurisdictions, and 19 tribes. These entities have, in turn, identified local communities to select and implement specific EBIs. In these types of initiatives, challenges and facilitators to EBI scale-up are likely to exist and interact across local, state, and federal levels.

**Table 1:** Structure of five public systems

System	Key outcomes	Federal agency(ies)	Structure
Behavioral Health	Mental, emotional, and behavioral (MEB) disorders, with a focus on mental health and substance use/abuse	Substance Abuse and Mental Health Services Administration (SAMHSA), US Department of Health and Human Services	Single state agencies (SSAs) and state mental health agencies (SMHAs) provide services; 70% of states combine these into one agency^1^
Child Welfare	Child maltreatment: safety, permanency, and well-being of maltreated children	Administration for Children and Families (ACF)	2/3 of states authorize local counties to provide services; 1/3 provide services at the regional level administered by the state. Some states (e.g., California) are a blend and operate as county administered but may have regional training centers
Education	Student academic achievement, truancy, graduation, discipline	U.S. Department of Education	Each state has a Department of Education, and areas of the state are organized into school districts to oversee education at the local level
Juvenile Justice	Juvenile crime	Office of Juvenile Justice and Delinquency Prevention	Each state has its own juvenile justice system that is regionalized in large states
Public Health	Physical, mental, and social well-being (WHO definition)	Department of Health and Human Services (DHHS), including the Centers for Disease Control and Prevention (CDC), Administration for Children and Families (ACF), the Health Resources and Services Administration (HRSA), the Substance Abuse and Mental Health Services Administration (SAMHSA), and the Office of the Assistant Secretary for Preparedness and Response (ASPR)	Each state has a single state health agency that works with local public health authorities (defined by county or city/town geography); 30% contain the single state agency for substance abuse; all contain a maternal and child health program

^1^According to the Substance Abuse and Mental Health Services Administration (2017)

We considered five public systems in this paper: behavioral health, child welfare, education, juvenile justice, and public health. We selected these systems because they have the greatest potential to intervene to prevent the majority of behavioral health problems^3^ by delivering EBIs at scale, with quality, and with significant reach in the US (National Research Council and Institute of Medicine 2009). Collectively, the five systems correspond to key developmental stages and ecological domains, making it theoretically possible for them to reach virtually all children, adolescents, and families with EBIs. The behavioral health, education, and public health systems provide universal, selective, and indicated services to all types of populations. The child welfare and juvenile justice systems implement primarily indicated interventions (e.g., services for youth alleged or substantiated as having experienced child maltreatment and/or to have engaged in illegal behaviors) with the aim of preventing further problems and/or engagement in these systems.

After forming the MAPS IV Task Force in 2016, we began reviewing available literature on EBI adoption, dissemination, and sustainability and discussing our professional experiences with scaling up EBIs. Following a series of conference calls and an in-person meeting, we drafted a preliminary list of challenges and facilitators we considered most important in scaling up EBIs in public systems. Because scale-up efforts will most often occur within, not across, systems (Cruden et al. 2016; Leslie et al. 2016), we then created five system-specific workgroups to consider these factors. Each workgroup was comprised of two to seven prevention researchers who had worked with practitioners and policy makers in these public systems. The workgroups then drafted system-specific papers or reports that identified the factors that were most important in their particular system, highlighted examples of successful EBI scale-up efforts, and provided system-specific recommendations to increase the scale-up of EBIs.

After this work was completed, it was clear that systems varied in their efforts and capacity to scale-up EBIs, and that the behavioral health, education, and public health systems seemed to be more successful than child welfare and juvenile justice. However, there was substantial overlap across systems in the factors that affected scale-up. The Task Force concluded that a paper that articulated these common elements could emphasize the magnitude and scope of the challenges that can impede scale-up, highlight strategies that some systems have used to enhance EBI scale-up, and facilitate *cross-systems collaboration*, which is important given the overlap in the outcomes and populations targeted by the five systems (Cruden et al. 2016; Leslie et al. 2016).

## The Factors That Affect EBI Scale-up in US Public Systems

**Fig. 1:**
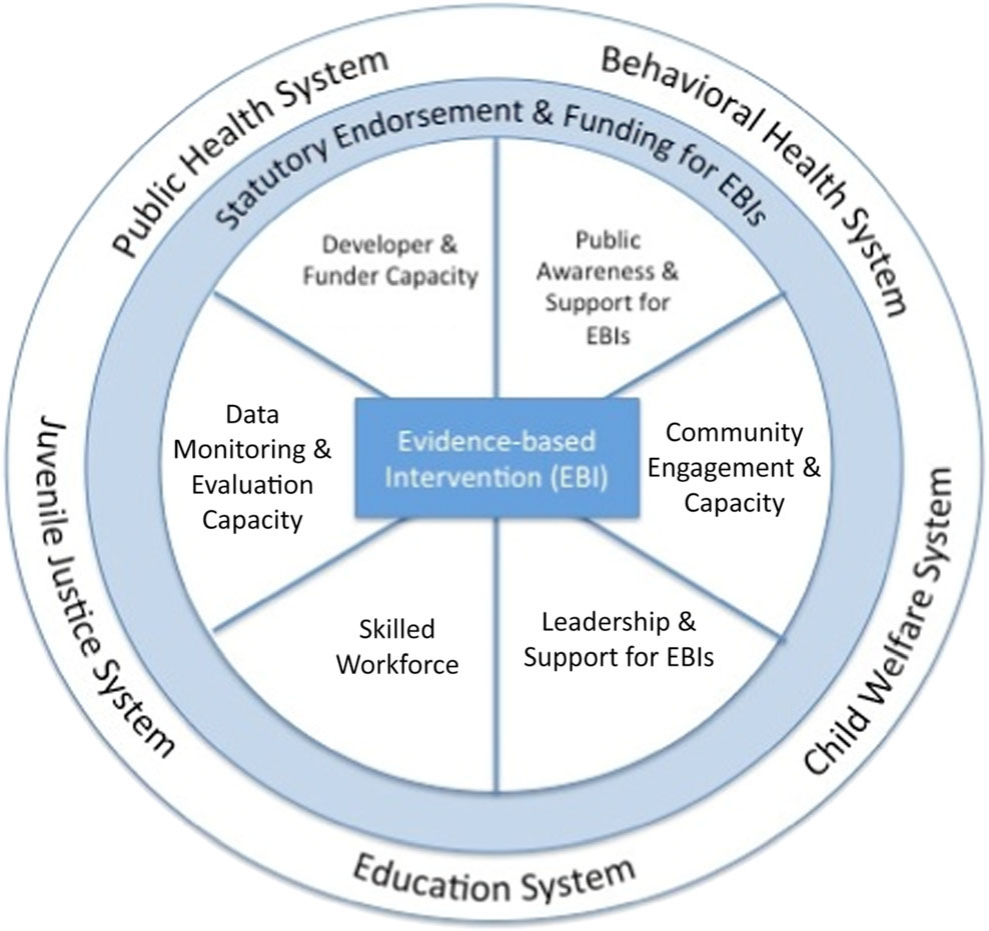
Ecological model identifying the factors that affect EBI scale-up in five public systems

Figure 1 illustrates the common set of factors that the MAPS IV Task Force and systems workgroups identified as affecting EBI scale-up across the five public systems. Figure 1 can be viewed either from the center outward (beginning with a focus on a specific EBI) or from the macro system(s) context and working inward to understand how the systems’ context and capacities impact the degree to which any EBI might be scaled up. As shown at the top of Fig. 1, we consider public policies (i.e., statutes, regulations, and guidance) and funding for EBIs as essential for scaling up. However, scale-up efforts also require developer and funder capacity; public awareness of and support for EBIs; community engagement and capacity; public systems leadership support for EBIs; a skilled workforce capable of delivering EBIs; and data monitoring and evaluation capacity.

Many of the factors shown in Fig. 1 were also identified as important by the MAPS II Task Force (Spoth et al. 2013) and in some implementation and dissemination literature reviews and frameworks (e.g., Aarons et al. 2017; Indig et al. 2018; Milat et al. 2015; Tabak et al. 2012). Nonetheless, our review indicated a very limited body of literature that has articulated specific actions or evaluated factors affecting EBI scale-up in particular, especially studies examining systems other than public health. Given the limited knowledge base, the following sections should not be understood as a definitive or comprehensive list of the factors related to EBI scale-up in public systems, but rather as a work in progress that requires further examination and evaluation.

## Statutory Endorsement and Funding for EBI Scale-up

In the US, public systems operate by following government directives and rely on public tax revenue to deliver services and enact policies. Given this reality, we consider EBI scale-up to be most influenced by the degree to which federal and state systems encourage EBI delivery and provide financial support for EBIs. As described in this section, in the last 10 years, the demand for and reach of EBIs has rapidly expanded through laws, regulations, policy guidance, and due to funding priorities that either mandate or encourage the use of EBIs. The illustrative examples discussed focus largely on federal initiatives and funds, given the strong influence the federal government has on state and local prevention activities, but some relevant state and community efforts are also described.

### Statutory Support for EBIs

Of the variety of mechanisms available to government agencies to endorse and fund EBIs, one of the most powerful and longest-lasting is the inclusion of language about the use of EBIs in federal and state statutes (Pew-MacArthur Results First Initiative 2015). The statute language may *require* a use of evidence when implementing services, *encourage* a use of evidence, and/or *allow* funds to be used for research, evaluation, or other aspects of the infrastructure necessary to create, implement, or scale-up EBIs (Pew-MacArthur Results First Initiative 2017).

Examples of federal statutes that have *required* EBIs have included the “tiered” evidence initiatives begun under the Obama Administration, such as the Maternal, Infant and Early Childhood Home Visiting Program (MIECHV) and the Teen Pregnancy Prevention Program (Haskins and Margolis 2014). The MIECHV legislation required that at least 75% of its designated funds be spent on early childhood home visiting models that had *demonstrated evidence of effectiveness*. The statute also had a provision that up to 25% of the funds could be spent on *promising approaches* as long as they were rigorously evaluated (Patient Protection and Affordable Care Act of 2010). A federal statute *encouraging* EBIs is the Personal Responsibility Education Program that required the adoption of an EBI or “substantially incorporated elements of” an EBI by agencies seeking to prevent high-risk sexual behaviors (Patient Protection and Affordable Care Act of 2010). As an example of the third type of statute, the Deficit Reduction Act of 2010 authorized grants for the Healthy Marriage and Responsible Fatherhood program as a demonstration program. The Act allowed funds to be used, in part, to conduct evaluations of the effectiveness of those programs, but it did not require the use of EBIs.

Some statutes provide very specific language concerning the study designs needed to determine effectiveness, for example, *randomized trials* (e.g., Patient Protection and Affordable Care Act of 2010), how long impacts must be sustained after programming has finished (e.g., the Family First Prevention Services Act Bipartisan Budget Act 2018), and the type of infrastructure required to provide enabling contexts for EBI implementation (Patient Protection and Affordable Care Act of 2010). The MIECHV statute, for example, requires implementing agencies to have “well trained staff” and a “program of continuous improvement” (Patient Protection and Affordable Care Act of 2010). Also, the Family First Prevention Services Act Bipartisan Budget Act of 2018 required the Secretary of DHHS to establish a clearinghouse of the practices that met the types of evidence specified in the Act.

States can also enact statutes and language to require, recommend, and/or support EBIs. Almost all states have some statute or regulation that funds EBIs in at least one major public system, and the majority of states define “evidence-based” based on some level of evaluation evidence (Pew-MacArthur Results First Initiative 2017). Some states have also passed statutes funding particular EBIs. For example, Colorado has a statute funding the Nurse-Family Partnership (Olds 2002) home visiting program (SECTION 1. Article 75 of title 24, Colorado Revised Statutes). In 2018, Utah passed legislation (H.B. 456 of 2018) that mandated the use of an evidence-based drug prevention curriculum in all schools (Dube and White 2017). This law resulted in the implementation of the LifeSkills Training program (Botvin and Griffin 2004) in every middle school because that program’s evaluation evidence most closely matched the statute’s requirements. More generally, a growing number of states are including specific line items in state budgets to fund EBIs and to promote the use of data and research evidence (White 2018).

### Regulations and Guidance

In addition to statutes, all levels of government agencies may use regulations and guidance to require or encourage EBIs. For example, the *Title V* Maternal and Child Health Block Grant has guidance that requires evidence-based practices to address national and state performance measures. These practices must be selected following a state needs assessment, and experts review the use of EBIs yearly for each block grant application (Kogan et al. 2015; Lu et al. 2015). Also, the Head Start Act of 2007 used the term “research-based” to describe interventions that could be implemented under this Act, but it defined this term only in the regulations released in 2017 (42 U.S.C. 9801 et seq., subchapter B of 45 CFR Chapter XIII). Additional guidance about the specific EBIs that met this definition was provided to grantees as policy guidance or technical assistance (https://eclkc.ohs.acf.hhs.gov/sites/default/files/pdf/ncedtl-ecc-research-based-curriculum.pdf).

In the education system, the Education Department General Administrative Regulations (EDGAR; https://www2.ed.gov/policy/fund/reg/edgarReg/edgar.html) provide definitions of varying evidence levels for grantees. In addition, non-regulatory guidance has been provided regarding the use of evidence in informing implementation of EBIs in conjunction with states’ implementation of the Every Student Succeeds Act (https://www2.ed.gov/policy/elsec/leg/essa/guidanceuseseinvestment.pdf). Similarly, the Individuals with Disabilities Education Act of 2004 (IDEA 2004) references the use of EBIs (i.e., “scientifically based instructional practices” and “research based interventions”) when outlining the permissible use of federal funds. Much of the guidance provided from the Department of Education builds upon and is largely consistent with the levels of evidence outlined in the *What Works Clearinghouse* (https://ies.ed.gov/ncee/wwc/).

Guidance in the form of Information Memoranda, “Dear Colleague” letters, and other formats also provide suggestions about which interventions can be implemented using public funds. However, because it is “guidance,” it is more challenging for the government to enforce these recommendations. For example, the Centers for Disease Control released a Dear Colleague letter in 2019 (https://www.cdc.gov/hiv/library/dcl/dcl/030819.html) to encourage the implementation of “proven” interventions to prevent HIV, but the recommendation did not provide detailed information about such EBIs and was not accompanied by any monitoring or enforcement.

Codifying the use of EBIs in statutory language has the greatest potential for scaling up EBIs because statutes are the most difficult to change once they are in place. Regulations and policy guidance are the second and third most difficult to change. Although statutes’ stability can be an advantage for scaling up, it can also be a disadvantage. For example, if statutory language does not mandate the use of EBIs, or if statutes are not aligned with the principles of prevention science, they may be less likely to improve public health and well-being.

### Funding for EBIs

The federal government generally issues funds to support EBIs through large, stable funding (e.g., *block grants*^4^) or smaller, shorter-term funding (i.e., *discretionary grants*). These two types of funding mechanisms also differ in how grantees are selected and the degree of flexibility in how funds can be used, both of which can affect EBI scale-up. Block grants are usually awarded to states through a non-competitive process, often based on a mathematical formula (https://blog.grants.gov/2016/06/15/what-is-a-block-grant/). The Department of Education’s *Title I* grants provide funding to local education agencies based on the number of children living in poverty (Golzalez and Tollestrup 2016). Block grants usually provide more funding than discretionary grants, but they tend to be more flexible and less restrictive, which can make it challenging for them to require EBIs. Nonetheless, there is some evidence that block grants can facilitate EBI scale-up. Some block grants (such as the *Title V* Maternal and Child Health block grant) have exclusively required EBIs for all funds provided, while others (such as the Substance Abuse Prevention and Treatment block grant) allow states flexibility to structure their prevention programs to meet local needs, which may include requiring a minimum set-aside for EBIs. Given the potential for block grant funding to support EBIs at scale and in the long-term, Liebman (2013) challenged the federal executive branch to allocate at least 5% of this type of funding to prevention via the implementation of EBIs.

Compared to block grants, discretionary grants are usually smaller in size. They are also competitive, as entities apply and are awarded funds based on eligibility and merit. Grants are usually time-limited (e.g., providing 3 to 5 years of funding) and have to be re-competed in subsequent cycles, which may reduce their utility for scaling up and sustaining EBIs. Discretionary grant funding is usually intended for a very specific purpose, as described in Funding Opportunity Announcements, but these mechanisms can stipulate that funds be used to implement EBIs. For example, the applications for SAMHSA’s Project LAUNCH (Linking Actions for Unmet Needs in Children’s Health) funded five types of EBIs: (1) home visiting, (2) family support and parent skills training, (3) mental health consultation in early care and education, (4) integrating behavioral health into primary care settings, and (5) developmental screening and assessment in child-serving settings.

Discretionary grants can foster innovation in EBI development and evaluation. In addition, the federal government has more direct control over the use of discretionary grants and can more easily require EBIs since the language and requirements in each request for proposals is crafted specifically for each announcement. However, as stated, discretionary grants are less able to ensure the sustainability of EBIs due to their limited size and duration. After a specific discretionary grant project period is complete, new grants are awarded on a competitive basis, so renewal of the funds to a specific entity is not assured.

Strategies to fund EBIs continue to evolve as the evidence base evolves. In the early stages of the field, discretionary and demonstration funding mechanisms were used to test the implementation of EBIs within longstanding public systems. To achieve scale-up, however, there has been an increased shift to the larger, more stable mechanisms that support services, such as the strategies described previously in relation to the Affordable Care Act and Families First Prevention Services Act, or through policy changes to Medicare and Medicaid services.

Funding through state and local tax authorities has also been used to fund EBIs. Some states and communities have mandated that a proportion of the dollars from tax levies (e.g., income or property tax) and excise taxes (e.g., on alcohol, tobacco and gambling) be earmarked for EBIs. For example, Washington State collects a 37% excise tax on retail sales of marijuana. In 2017, 10.7% of that tax revenue, or 27.8 million dollars, went to the state’s behavioral health agency to prevent and reduce substance use (Rushford et al. 2018), with 85% of this amount to be used for EBIs (Darnell et al. 2016). The Children’s Trust of Miami-Dade County (https://www.thechildrenstrust.org/) draws on real estate taxes to support EBIs to improve child health and well-being. County levies also have been used to support the implementation of specific EBIs, such as the Good Behavior Game in Ohio counties (Cruden et al. 2016).

In recent years, there has been a growth of Pay for Success to address the fiscal challenges of scaling up EBIs (Overholser 2018). In a Pay for Success project, the government contracts to obtain social services, and it pays (both for the costs of services and any additional profits) based on performance, which is evaluated based on evidence that agreed-upon outcomes have been reached. If outcomes are not achieved, the government does not pay. To date, many Pay for Success projects have been supported through Social Impact Bonds. Bonds are typically funded by philanthropic institutions and commercial investors who receive a share of the payments (Temple and Reynolds 2015).

Philanthropic foundations and corporations have also provided funding to implement EBIs in communities. Although foundation funding can be very helpful to local non-profit organizations operating with small budgets, most of these allocations are small, averaging about 20,000 dollars (https://philanthropynewsdigest.org/news/average-grant-sizes-increased-in-2016-foundation-source-finds). In addition, philanthropic funds tend to be short-term and allocated annually which reduce their utility for scaling up and sustaining EBIs (Roob and Bradac 2009). There have been larger investments, however, such as the Blue Meridian Partnership’s support for Wendy’s Wonderful Kids, and Edna McConnell Clark Foundation’s support of Nurse-Family Partnership. Another example, the Laura and John Arnold Foundation’s *Moving the Needle* solicitation, offered grants of up to five million dollars to support the scale-up of a small menu of evidence-based programs.

To summarize, federal, state, and local government, as well as philanthropic institutions and corporations, provide a variety of approaches to require, incentivize, and/or fund the use of EBIs. These mechanisms vary in their utility for scaling up EBIs. Public policies that require rather than recommend the use of EBIs likely having the greatest potential to enhance EBI scale-up in public systems. To do so, these statutes should clearly identify the interventions considered to be EBIs (and ensure that they have been subject to rigorous evaluation), and government and other entities should provide a coordinated set of funding options (e.g., block grants and discretionary funds) that include stable funding streams to facilitate scale-up once interventions have established a certain level of evidence. Given public investments in EBIs, economic analyses should be conducted before, during and after EBI scale-up (Crowley et al. 2018; Crowley et al. 2014). Resources, infrastructure, and oversight are also necessary to support the high-quality implementation of EBIs and ensure accountability.

## Developer and Funder Capacity to Scale up EBIs

Although a growing number of EBIs have been developed, tested, and demonstrated to be effective over the last few decades, many were not originally developed with scale-up in mind, particularly those created when prevention science was just emerging as a scientific discipline. Traditionally, most evidence-based programs and some practices have been created using a phased approach in which scientists create interventions based on theories and prior research regarding hypothesized risk and protective factors that influence behavioral health problems, then test these hypotheses by evaluating interventions in efficacy trials (Institute of Medicine 1994). In some cases, developers or other researchers then examine EBI effectiveness in larger-scale trials involving larger and more diverse populations and more “real-world” conditions (Flay et al. 2005; Gottfredson et al. 2015). However, most research ends at this stage, resulting in a lack of knowledge about scale-up.

Critics contend that this phased approach results in interventions that are not suitable for scale-up because they lack external validity and/or consumer appeal (Glasgow and Emmons 2007; Glasgow et al. 2003; Rotheram-Borus and Duan 2003). EBIs developed in this manner may be too complex to be implemented in systems, they may require a level of knowledge and skills that most of the workforce does not have, and the populations with whom they were tested may not resemble typical clients served by public systems (Dodge 2018; Supplee and Metz 2015). In addition, if developed without input from the participants they will serve, EBIs can contain content or use methods of delivery that are not engaging or culturally relevant or appropriate for participants (Barrera Jr. et al. 2017). For example, most parent training programs include attention to child discipline strategies, but parents from different racial/ethnic groups are likely to vary in their support for and willingness to use certain disciplinary methods (e.g., time-out or spanking) (Forehand and Kotchick 1996).

Especially in the formative era of prevention science, during which many currently recognized EBIs were first developed, the mismatch between EBI development and scale-up may have occurred because scientists were focused on experimental etiology; that is, using principles from theories of human development to create programs that they hypothesized would change participant behavior. Once they had tested the intervention and confirmed their hypotheses, these scientists may not have had the time, skill, financial/human resources, or desire to scale up their interventions. Further, most program developers were trained in traditional scientific methods of research and evaluation, but perhaps not program implementation, quality improvement, dissemination, management, marketing, or communication (Eddy et al. 2005; Kreuter and Bernhardt 2009; Stamatakis et al. 2013). Although there has been an increasing awareness of the need to design EBIs with scale-up in mind, some program developers still may have insufficient knowledge and/or contact with the systems that will implement the EBIs or the users who will experience them, and there are still few incentives for developers to engage in scale-up efforts (Neuhoff et al. 2017). Until recently, relatively little funding has been allotted for effectiveness and dissemination trials and few journals published articles focused on implementation and dissemination research (Glasgow et al. 2012).

All of these barriers limit the degree to which existing EBIs are ready for scale-up. However, with the increasing focus on EBI scale-up, the current generation of program developers are more likely to utilize an approach of *designing for diffusion* (Dearing and Kreuter 2010) when creating, evaluating, and scaling up EBIs. Rather than moving research into practice, as in the traditional approach, scientists include input from EBI users and implementers from the outset and create “pull” from consumers, rather than “push” interventions into use. Similarly, it is important to create two-way communication and engagement between research, practice, and policy partners to co-design interventions (Tseng et al. 2017). The goal is to ensure that policy and practice shape research agendas and to create feedback loops that will help improve the effectiveness and scale-up of EBIs (Bradshaw and Haynes 2012; Palinkas et al. 2015; Tseng et al. 2017). Boothroyd et al. (2017) suggest that *active involved community partnerships* between scientists and community members are pivotal in co-creating the implementation infrastructure needed to achieve and sustain health outcomes in scale-up efforts. In fact, some funding opportunities (e.g., from the Patient-Centered Outcomes Research Institute, PCORI) now include requirements for community representation. Additionally, there have been calls to create state-level inter-systems and inter-departmental backbone organizations, such as executive level “Children’s Cabinets” that can provide a unified vision, financial resources, accountability, and technical assistance across systems (Gaines et al. 2017; Hawkins et al. 2015; National Opinion Research Center (NORC) 2011; Rhoades et al. 2012).

Recognizing the obstacles that limit scientists’ abilities to market their own EBIs, Kreuter and Bernhardt (2009) call for *purveyor* and *intermediary organizations* to promote EBIs and their scale-up. Purveyor organizations are for-profit companies or non-profit organizations devoted to helping potential users learn about and successfully implement and sustain specific EBIs (Franks and Bory 2015; Neuhoff et al. 2017). For example, developers of Nurse-Family Partnership, LifeSkills Training, and Triple P Positive Parenting Program have all created purveyor organizations to provide community-based organizations with training and technical assistance to implement these EBIs. Intermediary organizations are usually not affiliated with a particular EBI, but rather assist organizations and systems to select and implement a variety of EBIs (Fixsen et al. 2005; Franks and Bory 2015). For example, the Collaborative for Academic, Social, and Emotional Learning (CASEL) provides schools with guides (Collaborative for Academic, Social, and Emotional Learning (CASEL) 2013, 2015) and electronic resources (https://drc.casel.org/) to help them learn about and select appropriate EBIs intended to promote students’ social and emotional learning.

Purveyor and intermediary organizations may have more time, expertise, and incentives to conduct adequate consumer research, effectively brand and package EBIs, distribute them, and provide training and technical assistance to support implementation in public systems (Kreuter and Bernhardt 2009; Supplee and Metz 2015). They may also be better positioned to understand the context of public systems and propose policy or practice changes that enable public systems to adopt EBIs. For example, Chamberlain (2017) described how her purveyor organization worked with administrators of child welfare systems in New York and Tennessee to scale up two family-focused EBIs. The organization collaborated with the New York Administration for Children and Families (ACS) to have caseworkers implement the EBIs instead of referring parents to other organizations, which meant the purveyor also had to work with ACS to identify ways to reduce caseworkers’ workloads to accommodate this shift in responsibilities. Another purveyor organization, the National Center for Positive Behavioral Interventions and Supports (PBIS), develops and disseminates free tools via its website (PBIS.org) to support high fidelity implementation of the PBIS framework and related EBIs at school, district, and state levels.

Although some purveyor organizations have partnered with public systems and agencies to help them select and implement EBIs, a survey of 46 purveyor groups that worked with child welfare and juvenile justice systems indicated that most currently lacked the capacity to scale-up EBIs (Neuhoff et al. 2017). Like many scientists, most of these purveyor organizations reported having a limited number of staff, insufficient funding, and a lack of marketing expertise which together impeded their ability to promote EBIs at significant scale (see also Franks and Bory 2015). The survey found that 60% of the organizations served less than 10,000 individuals per year, a figure that may be too small to result in population-level improvements in health and well-being, especially if that population is spread over many states.

At this point, there is little consensus on the optimal role, responsibilities, or even the feasibility of relying on researchers to take EBIs fully from theory to scale. There is, however, agreement that a more comprehensive research and development infrastructure is necessary to scale-up interventions that demonstrate strong evidence of effectiveness. For example, the research-to-dissemination pipeline may need to be expanded with more input from practitioners, policy makers, and consumers. Research and funding is also needed to “optimize” existing evidence-based programs and practices (Bumbarger 2015) and help them become more transportable and scalable. Such efforts might focus on identifying or adapting the core components in order to create a “leaner” (i.e., shorter and easier to deliver) intervention that is more feasible to scale-up (as recommended in HRSA’s Home Visiting Research and Development Platform; see https://www.hvresearch.org/). It is also important to examine if and how adaptations made during the scaling up process, especially those made to accommodate systems’ needs and resources, affect EBI outcomes. Positive and supportive partnerships between EBI developers, practitioners, and policy makers will be needed throughout these stages of development, evaluation, and scale-up (Palinkas et al. 2015; Spoth et al. 2013; Supplee and Metz 2015).

## Public Awareness of and Support for EBIs

The statutory supports and funding provided by government and philanthropy are essential for scaling up EBIs (Milat et al. 2015), but top-down approaches alone are insufficient for achieving the level of scale-up needed to produce population-wide improvements in health and well-being (Hawkins et al. 2015; Rhoades et al. 2012; Spoth et al. 2013). There must also be strong support for EBIs among the public and from systems administrators and staff (Aarons et al. 2011; Damschroder and Hagedorn 2011; Metz and Bartley 2012). As Hawkins et al. (2015) indicate, fully “unleashing the power of prevention” requires a large-scale shift in public attitudes about evidence-based prevention, such that the general population becomes more aware of EBIs and demands that they be scaled up. Scaling up thus requires not only a *supply* of EBIs (and funding to support their implementation), but also *demand* for them.

To increase demand, information about EBIs must be better communicated to the public and to those working in the public systems where EBIs are delivered (Brownson et al. 2018; Spoth et al. 2013; Wandersman et al. 2008). Progress has been made in this area, particularly with the development of “what works” registries that provide information about EBIs^5^ in accessible outlets and user-friendly formats. Public systems have created and manage some of these registries to identify EBIs that are required or recommended by public statutes and/or guidance. For example, the Department of Education’s *What Works Clearinghouse* (https://ies.ed.gov/ncee/wwc/) describes school-based EBIs, the Office of Juvenile Justice and Delinquency Prevention’s *Model Programs Guide* (https://www.ojjdp.gov/mpg) identifies EBIs that prevent delinquency, and the Centers for Disease Control lists public health EBIs on its *Community Guide* (https://www.thecommunityguide.org/). These registries require regular updating, maintenance, and sustained investment by the agencies in order to remain relevant, timely, and user-friendly (Goesling et al. 2017).

Although registries have helped raise awareness of EBIs (Baum et al. 2013; Hallfors et al. 2007; Neuhoff et al. 2015; Novins et al. 2013), systems leaders and staff often struggle to select EBIs from these lists. Feedback from child welfare and education administrators indicated that they wanted registries to provide more than information about EBI effectiveness (Neuhoff et al. 2015). They also wanted to know about EBI implementation requirements, costs, targeted populations, and targeted risk and protective factors. Although there are some exceptions, most registries do not provide this information (Avellar et al. 2017; Horne 2017; Neuhoff et al. 2015). In addition, registries typically do not typically provide much guidance on adaptation, but this is also important information when taking EBIs to scale (Aarons et al. 2017).

These examples illustrate that more effort is needed to increase awareness and support for EBIs and to help public systems leaders and staff select EBIs that can be feasibly implemented and successfully scaled up. Intermediary organizations offer a promising avenue for increasing EBI scale-up in public systems, particularly state-level *Centers of Excellence* that offer support to systems leaders and staff across an entire state (Hoagwood et al. 2014; Mettrick et al. 2015). For example, the Institute for Innovation and Implementation at the University of Maryland (https://theinstitute.umaryland.edu) provides training, technical assistance, and workforce development initiatives to agencies across the state to support a variety of early childhood, child welfare, and juvenile justice EBIs. Likewise, the Evidence-based Prevention and Intervention Support Center (EPISCenter; http://www.episcenter.psu.edu/) at the Pennsylvania State University builds the capacity of community coalitions across the state to select and monitor implementation of EBIs listed on the Blueprints for Healthy Youth Development registry (https://www.colorado.edu/cspv/blueprints/) (Rhoades et al. 2012).

State-level intermediary organizations can assist systems level scale-up in several ways. They can organize centralized trainings for staff delivering the same EBI across the state, rather than have each organization pay for its own training, which may save money and create communities of learners who can support one another (Mettrick et al. 2015). Similarly, a state-level intermediary may pay for its staff to become certified to provide trainings and/or technical assistance in an EBI(s) and may also pay licensing fees to EBI developers to allow for implementation, which can also defray the costs otherwise incurred by smaller local agencies. Although intermediary organizations show promise for increasing scale-up, they have a relatively short history and to date there has been little empirical evaluation of their ability to foster EBI scale-up.

## Community Engagement and Capacity

Increasing awareness of EBIs and building the capacity of systems to select and implement EBIs should generate more demand for EBIs and increase the political will for scale-up (Milat et al. 2015; Richmond and Kotelchuck 1983). Advocacy for EBI scale-up can be done at multiple levels, but the active engagement of community leaders and stakeholders is essential (Milat et al. 2015). While there may be federal or state mandates to implement EBIs (Sheras and Bradshaw 2016), local communities will vary in the specific causes of behavioral health problems, and they may need different EBIs to address these needs (Catalano et al. 2012; Spoth et al. 2013; U.S. Department of Health and Human Services, Office of the Surgeon General 2016). Local community members are well positioned to inform and advocate for the selection of the EBIs that will be most feasible to enact and most responsive to their needs, resources, culture, and norms (Spoth et al. 2013). They can also suggest potential adaptations to EBIs to increase the “fit” of the intervention to the local community and its diverse cultures (Castro et al. 2010; Castro et al. 2004). Reliance on members of the local community to select, implement, and monitor EBIs should enhance residents’ investment in and support for evidence-based prevention, particularly if such actions can show that interventions are producing their intended effects on the outcomes prioritized by the community. These efforts should, in turn, improve the quality of implementation, foster accountability, and increase the likelihood that interventions will be sustained (Foster-Fishman et al. 2007; Wallerstein and Duran 2010).

Some public systems have actively promoted community engagement in EBI implementation and scale-up. For example, most federal public health grants (e.g., Healthy Start and REACH) require community advisory groups or consortia. The Maternal and Child Health Services *Title V* program has long included parent, family, and community partners and most states have a “family representative” on their staff. In addition, the Affordable Care Act (ACA) community benefits approach requires non-profit hospitals to obtain community input when setting and addressing public health priorities (Nelson et al. 2013). SAMHSA’s Strategic Prevention Framework (https://www.samhsa.gov/capt/applying-strategic-prevention-framework), which promotes and funds EBI scale-up in behavioral health, relies on broad-based community coalitions to assess community needs, collectively agree on EBIs that best address their needs, and monitor and evaluate EBI implementation. Coalitions should include representatives from multiple public systems (e.g., education, juvenile justice, public health) so they can promote a coordinated delivery of multiple EBIs targeting multiple risk and protective factors shared across diverse behavioral outcomes.

The ability of community-based coalitions to increase EBI use and reduce behavioral health problems has been demonstrated in evaluations of the CTC and PROSPER coalition systems. These studies have shown that these systems foster the adoption of EBIs and reduce youth substance use and delinquency among entire age-grade cohorts in intervention compared to control communities (Hawkins et al. 2009; Hawkins et al. 2012; Spoth et al. 2011; Spoth et al. 2015). In addition, a statewide scale-up of CTC in Pennsylvania demonstrated statewide reductions in youth substance use and delinquency and improvements in academic performance (Feinberg et al. 2007; Feinberg et al. 2010). These examples not only illustrate the value of engaging communities in EBI scale-up, but also suggest “tipping point” effects wherein a larger population may benefit when a sufficient proportion of that population is exposed to EBIs. However, evaluations of CTC and PROSPER also indicated that communities require sufficient funding, leadership, support and capacity to select, monitor, scale-up, and sustain EBIs. Coalitions have also been successfully engaged in public health; for example, in a Massachusetts tobacco control initiative that created smoke-free restaurants and workplaces, increased the price of tobacco, and enforced prohibitions restricting sales of tobacco to minors (Koh et al. 2005).

## Public System Leadership and Support for EBIs

Public systems leaders are critical to the successful scale-up of EBIs (Novins et al. 2013). A review of factors associated with the successful scale-up of ten health promotion or disease prevention EBIs found that leaders who were “invested, dedicated, and interested were instrumental in facilitating implementation and sustainability” (Norton and Mittman 2010, p. 21). Similarly, a review of public health EBIs found that strong leaders were one of the most frequently reported facilitators of scale-up (Milat et al. 2015). According to Welsh and Greenwood (2015), states that have been most successful in scaling up juvenile justice EBIs had committed key leaders at the highest levels of the state administration (e.g., state governors and commissioners of the corrections system). These leaders facilitated scale-up by generating public support and funds for EBIs and communicating the value of EBIs over business as usual.

Leaders are essential because they have the decision-making authority to determine whether or not EBIs should be endorsed, adopted and sustained, and they have access to the funds necessary to support proper implementation (Aarons 2006; Damschroder et al. 2009). They are also important in engendering support for EBIs from others, especially the staff charged with delivering EBIs (Aarons 2006). According to Aarons (2006), Aarons et al. (2016), leaders who articulate how EBIs will help address organizational goals and needs and who provide tangible rewards and positive reinforcement to EBI implementers engender more positive attitudes about these interventions among staff.

Conversely, a lack of supportive leadership is a major barrier to EBI implementation and scale-up (Milat et al. 2015; Norton and Mittman 2010). Leaders who are resistant to innovation and comfortable with the status quo can impede dissemination efforts (Brownson et al. 2009). Moreover, those who lack knowledge about the existence or advantages of EBIs will not be able to generate support for them. Loss of effective leaders through turnover, including political cycles, is also a common challenge in public systems. The average duration of school district superintendents is about 6 years (The Broad Center 2018). In the child welfare system, elected officials typically appoint leaders (e.g., Secretaries and Directors), meaning they can change with elections. Similarly, in public health, the state public health director is a cabinet level position in 53% of states, making these leaders vulnerable to turnover (Beitsch et al. 2006).

Scaling up EBIs requires efforts to educate leaders about EBIs, build their support for prevention, and minimize or mitigate the effects of turnover as much as possible. These actions must take into account the hierarchical nature of leadership; that is, the fact that leaders exist at multiple levels (e.g., community, state, and federal) in the system and that leaders at each level vary in their decision-making authority and influence. This variation makes it difficult to ensure that EBIs are consistently scaled up within a system. Even if top leaders are familiar with EBIs and support their scale-up, a lack of knowledge and support from local leaders may inhibit EBI scale-up. Further, public systems that primarily serve referred or selected populations (e.g., child welfare and juvenile justice) may find it challenging to prioritize prevention if doing so is seen as competing for resources to serve their already system-involved populations.

Yet there are some promising solutions to build leadership knowledge of and support for EBIs. In child welfare, the National Child Welfare Workforce Institute’s Leadership Academy for Deans and Directors (LADD) pairs deans and directors of university-based social work programs with child welfare directors (see: https://ncwwi.org/index.php/teams-services/ladd). The LADD helps each pair develop a university-agency partnership and identify an EBI that can be taught to graduate students at the university and then implemented in child welfare practice settings. In behavioral health, the National Association of State Alcohol and Drug Abuse Directors (NASADAD) created the National Prevention Network (NPN; http://nasadad.org/npn-4/) to increase leaders’ understanding of prevention issues and help them engender support for prevention among their workforce and communities. Leadership training to improve public health practice is available to state and local public health workers through a variety of leadership institutes available at the national level and in many states (https://www.cdc.gov/stltpublichealth/nlaph/index.html). The CDC provides support and partnership with the National Leadership Academy for the Public’s Health (NLAPH) (http://healthleadership.org/program_nlaph).

## A Skilled EBI Workforce

One of the reasons that leaders are essential to EBI scale-up is because they oversee the staff who are charged with delivering, supporting, and/or monitoring these interventions. In this regard, system leaders determine whether or not the system/organization has an adequate number of stable staff to promote widespread delivery of EBIs, as well as a workforce that is ready, able, and excited to fully implement these interventions. According to literature reviews of EBI scale-up in public health (Milat et al. 2015) and behavioral health (Novins et al. 2013), staff support is an important facilitator of success, and a lack of human resources and poor investment in staff training are significant barriers to EBI scale-up. The US Surgeon General’s report on alcohol, drugs, and health (U.S. Department of Health and Human Services, Office of the Surgeon General 2016) also identified limited workforce capacity as a key challenge to preventing substance use disorders. The report noted that many frontline staff do not understand the risk and protective factors related to substance use or that there are EBIs that address these factors (U.S. Department of Health and Human Services, Office of the Surgeon General 2016).

There must be an adequate number of staff to reach enough participants with services to reduce behavioral health problems and improve well-being at a population level. Staff may also need certain educational or experiential credentials in order to deliver particular EBIs. The expectation is that staff with these credentials will already have the knowledge and skills needed to deliver EBIs. The reality is that professional development and credentialing organizations rarely provide specialized information about EBIs and EBI implementation, which leaves staff ill prepared to support or deliver EBIs. A SAMHSA (2017) survey supports this statement, as 96% of state mental health agencies rated staff readiness as “sometimes” and “always” a barrier to EBI implementation.

In the juvenile justice system, staff (e.g., juvenile probation officers) have traditionally viewed their primary role as supervision and surveillance, rather than as service brokers or interventionists. As research has emerged indicating that therapeutic interventions are more effective than surveillance in reducing delinquency (Lipsey 2009; Lipsey et al. 2010), system leaders recognized the need to re-orient and re-train their workforce to deliver prevention services. Likewise, in child welfare, the primary role of staff (i.e., caseworkers) has traditionally been case management (e.g., assessing family needs and making referrals to other agencies for services) rather than service delivery, which means that training programs did not include education on EBI delivery. With the passage of the Families First Prevention Services Act, many state systems expanded staff responsibilities to include interventionist activities, making pre-service EBI training more important. In the education system, teacher preparation programs have not usually included information on EBIs. According to a large-scale study of teachers, many reported that they were not well equipped to intervene when they observed bullying behaviors (the focus of the survey), and nearly three-fourths stated that they could benefit from training on how to respond to bullying (Bradshaw et al. 2011). Moreover, teachers generally lack knowledge about EBIs, particularly in relation to the promotion of positive student behavior and well-being (Stormont et al. 2011).

Staff disciplinary backgrounds, educational level, and familiarity with EBIs vary significantly within systems. As a result, it can be difficult to ensure consistent standardization and quality of EBI delivery. Acknowledging these deficits in the workforce, most evidence-based programs and practices require or strongly recommend that implementers receive on-the-job training to learn about the intervention’s effectiveness, understand the core components that lead to such changes, and acquire the skills to deliver it with quality (Becker et al. 2013; Durlak and DuPre 2008; Dusenbury et al. 2003; Fixsen et al. 2005). Training also promotes staff self-efficacy—the belief that one can successfully implement an EBI—and support for the EBI, each of which can improve EBI implementation quality and effectiveness (Beets et al. 2008; Bradshaw et al. 2018; Damschroder et al. 2009; Durlak and DuPre 2008).

Although training is important, staff may not receive it, particularly in systems like education where there is limited time and competing demands for professional development training. Even when implementers are qualified and trained to deliver EBIs, they may not reside in the system long enough to facilitate EBI scale-up. Staff turnover remains a major challenge to the effective implementation and scale-up of EBIs across systems (Milat et al. 2015; Norton and Mittman 2010; Novins et al. 2013; Spoth et al. 2013). In child welfare, high caseloads and the emotional toll of interacting with children and families lead to frequent turnover (Lizano and Mor Barak 2012). Loss of staff who have been trained to deliver EBIs means that systems will have to spend additional time and resources to train new staff.

Public systems have developed some promising workforce development training approaches that may help address these challenges and build workforce capacity to deliver EBIs. The Title IV-E Education and Training program is a federally funded initiative in about 70 social work programs that prepares students interested in child welfare (Zlotnick and Cornelius 2000). In addition, every MSW program requires students to complete at least 900 hours of practical experience in social service agencies, which provides an opportunity to familiarize students with implementation of some EBIs. The American Professional Society on the Abuse of Children (APSAC) has recently developed practice guidelines to assist child welfare workers with conducting evidence-based service planning (EBSP), which involves developing service plans and selecting effective treatments (https://apsac.memberclicks.net/practice-guidelines). In public health, a voluntary Certified in Public Health exam administered by the National Board of Public Health Examiners (https://www.nbphe.org/) can ensure that staff have required competencies. In behavioral health, Prevention Specialist Certification programs are available in many states under the International Certification and Reciprocity Consortium to provide training to staff in EBI delivery.

Across systems, it is important to ensure that the workforce has adequate cultural competency targeted to their populations (Barrera Jr. and Castro 2006). Staff who possess such competencies are more likely to recognize, understand, and respect individual and group differences. They are also more likely to use this knowledge to adapt their delivery styles to meet the needs of their clients or students, especially those from minority groups (Gregory et al. 2012). Examples of resources for building cultural competency include the Center for Community Collaboration (CCC), located at the University of Maryland, which provides such training for behavioral health system staff (Gregory et al. 2012). Nationally, SAMHSA’s Strategic Prevention Framework (https://www.samhsa.gov/capt/applying-strategic-prevention-framework) emphasizes the importance of cultural competence when selecting and implementing EBIs to prevent substance use/abuse. SAMHSA provides communities with training, materials, and technical assistance to help them fulfill this mandate.

Staff competencies may also be enhanced through receipt of on-going supervision and coaching (Pas et al. 2014), and these supports are especially important when staff enter the workforce with a deficit of training in EBIs (Bradshaw et al. 2018). Within systems, supervisors should oversee EBI delivery and provide supportive and formative feedback to staff (Johnson et al. 2018). Some dissemination literature advocates that evidence-based programs and practices be overseen by *implementation teams* comprised of multiple implementers as well as their supervisors and/or administrators and even community members (Fixsen et al. 2013; Newton et al. 2011). These implementation teams are conceptualized at the local level (i.e., within a service provider organization), but creating larger learning communities across organizations in a system is also useful for information-sharing, group problem solving, and social support (Flaspohler et al. 2012; Hawkins et al. 2015; Norton and Mittman 2010; Novins et al. 2013).

Coaching and technical assistance may also be provided by program developers and/or intermediary organizations. Such support can help ensure that staff are implementing EBIs fully, with quality, to the populations for whom they are intended, and with a reach sufficient to produce population-level changes (Damschroder et al. 2009; Fixsen et al. 2005; Spoth et al. 2013). In fact, many EBI developers *require* that agencies obtain technical assistance from them, so that they can ensure that EBIs are replicated with consistency and fidelity across sites. However, it is important to keep in mind that systems that are under-resourced may struggle to purchase on-going technical assistance.

## EBI Data Monitoring and Evaluation Capacity

Prevention science research indicates that EBIs are most effective when they are delivered as intended and that they may be ineffective when poorly implemented (Durlak and DuPre 2008; Fixsen et al. 2005). There is also evidence that adaptations are commonplace (Cooper et al. 2016; Moore et al. 2013) and that some changes may be beneficial, such as when they help engage participants and/or enhance the fit of the EBI with the local context (Barrera Jr. et al. 2017; Hall et al. 2016; Huey et al. 2014). Systems must develop a culture that values and prioritizes the collection, analysis, and use of data on EBI implementation and outcomes to track both the changes made and their impact on participants (Bradshaw et al. 2012).

Implementation data can provide information on how well services are being delivered, the degree to which implementation goals are being met (including the number and types of participants served), and the degree to which desired outcomes are being realized. If collected systematically and regularly analyzed, the data can identify implementation challenges early on, before they threaten EBI success (Blase and Fixsen 2013; Moore et al. 2013; Proctor et al. 2013), as long as staff and/or administrators take corrective actions to address the challenges.

Collecting and analyzing data on participant outcomes can help determine the degree to which EBIs are changing targeted risk and protective factors, reducing behavioral and other health outcomes, and promoting positive outcomes. Being able to demonstrate positive effects can help maintain and/or increase staff and leadership support for EBI implementation and may promote additional efforts to scale-up and “scale out” EBIs in new settings and populations (Aarons et al. 2017). Alternatively, a lack of positive results may indicate that the EBI has not been well implemented and that improvements to its delivery must be made. Continuous evaluation may also inform the choice to de-adopt (Niven et al. 2015) a particular EBI and replace it with another EBI that may be more feasible to implement or better suited to the context.

Both short- and long-term outcome data should be collected because outcomes may not be realized until months or years after EBI implementation (Farrington and Welsh 2013; Gottfredson et al. 2015). For example, caregivers may participate in parent training interventions when children are in early elementary schools, but improvements in children’s externalizing or internalizing behaviors and/or substance use may not be evidenced until middle or high school (Sandler et al. 2015). Some federal and state systems (e.g., State Longitudinal Data Systems in education) have been proactive in building integrated data systems that allow the tracking of individuals over time (Pew-MacArthur Results First Initiative 2017). Although privacy issues must be attended to, these databases can identify participation in various EBIs and ideally allow the linkage of outcome data across time and systems (i.e., *data interoperability*) to fully assess EBI effectiveness. The Balanced Budget Act of 2018 included language that directed agencies to work on data interoperability across and within federal programs. The Bipartisan Commission on Evidence-Based Policy Making also notes that integrated data systems can inform public investment in EBIs (https://www.cep.gov/content/dam/cep/report/cep-final-report.pdf).

Having adequate implementation and outcome monitoring systems in place was one of the most frequently mentioned facilitators of public health EBI scale-up in the review by Milat et al. (2015). However, systems vary in their capacity to adequately monitor implementation, analyze implementation and outcome data, and make changes to EBI delivery to address identified challenges. These systems can be costly to create and maintain, especially when they require hiring and/or training staff, and these costs may or may not be supported through funding mechanisms that support EBI implementation.

All five of the systems included in this review have some procedures in place to collect, record, and analyze data, but they vary in their ability to collect data on EBI implementation and on short-and long-term outcomes at an individual or systems level. In the behavioral health and public health systems, outcomes are routinely tracked, with data typically collected and managed at the state level through multiple population-based surveillance systems. Examples include Vital Birth and Death Records, Cancer Registry, Pregnancy Risk Monitoring System, and statewide surveys to monitor health behaviors (e.g., the Behavioral Risk Factor Surveillance System) and to assess school climate and students’ behavioral problems and exposure to risk and protective factors. In addition, many public health systems have mechanisms in place to collect and analyze data on EBI delivery, and they use these data for continuous quality improvement.

In child welfare, federal mandates stipulate that state systems report performance outcomes related to child safety, permanency of children’s living arrangements, and children’s well-being. Quantitative data on outcomes are supplemented by qualitative data obtained through staff site visits and case reviews, which often examine indicators of the quality of EBI and other services. In states that are county-administered, data tracking programs may vary by county, making it difficult to link the data across counties to obtain state-level results. Another problem is that these systems are not well equipped to collect data on EBI delivery provided by partner agencies and/or behavioral changes in participants following receipt of services. Better data systems are needed that are flexible enough to capture implementation data across EBIs, yet structured enough to allow for easy input and analysis by child welfare workers. Ideally, these systems could be also linked to other administrative records, including those collected in other systems (e.g., juvenile justice).

The education system also has barriers and facilitators to data collection and analysis. Data are collected on the academic progress of every student in every public school, as well as student and school-level outcomes that may be affected by EBIs (e.g., truancy and drop-out rates). However, schools and districts do not typically collect information on specific EBIs delivered in classrooms or at the school level, and linking data across school districts is not common. Many states have data systems to track information on students’ receipt of special education services, which, consistent with the Individuals with Disabilities Education Act (IDEA), are expected to include receipt of EBIs. But school and district personnel may lack the skills needed to reliably enter and analyze implementation or outcomes data for other types of EBIs, and to do so in an efficient and timely manner so that problems can be quickly resolved. Some frameworks for implementing EBIs, like PBIS, strongly encourage the use of data systems by schools, districts, and states to track not only local implementation of PBIS and related EBIs, but also to monitor EBI outcomes (e.g., Bradshaw et al. 2014). Several tools have been developed to support PBIS monitoring and EBI fidelity and outcomes, such as the School Wide Information System (SWIS.org) for tracking discipline referral data (Irvin et al. 2006).

In the juvenile justice system, Walker et al. (2015) emphasize the need to improve states’ “diagnostic and evaluative capacity.” They recommend the creation of comprehensive systems that include needs assessment data to facilitate EBI selection and implementation and outcome data to allow continuous quality improvement. Although some states have made progress in doing so, others have not.

These challenges indicate the need for financial and other supports to system administrators and staff to facilitate the collection and evaluation of EBI data. Some intermediary organizations have provided such assistance in particular systems. A survey of 68 intermediary organizations in health, behavioral health, and education indicated that a primary function of three-fourths (76%) of the agencies was helping organizations and/or state agencies to collect implementation data and conduct continuous quality improvement (Franks and Bory 2015).

## Discussion and Conclusion

As articulated in this paper, public systems are essential for scaling up EBIs because they provide the infrastructure and resources necessary to enact these prevention policies and services (also see Brown and Beardslee 2016). Moreover, the systems operate at federal, state, and local levels, and are thus the *only* way to reach the entire population of youth, families, and communities with EBIs. Yet Patrick McCarthy (2015, p. 13), former President of the Annie E. Casey Foundation, has noted that “a bad system will trump a good program every time.” Although this is an overstatement, the MAPS IV Task Force discussed at length the challenges inherent in public systems that have hindered or failed to facilitate EBI scale-up. Nonetheless, we also identified many factors present within and across five public systems (i.e., behavioral health, child welfare, education, juvenile justice, and public health) that have increased EBI scale-up. In particular, federal agencies overseeing major public systems in the US have set statutory requirements that states and communities implement EBIs and provided public funds to do so. These actions have greatly facilitated the scale-up of some EBIs.

For these reasons, when charged with the task of identifying ways to scale-up EBIs to improve population health and well-being, we chose to focus on the factors inherent in public systems that can facilitate and/or impede scale-up. This work built upon the efforts of the Type II Task Force (Spoth et al. 2013), which also acknowledged the importance of government funding and support for EBIs and identified some of the same infrastructure requirements for EBI implementation (e.g., leadership, workforce development, and EBI data monitoring systems) as the current paper. We also drew upon prior literature examining the numerous political, organizational, individual, and intervention-level factors that affect EBI dissemination and implementation (e.g., Milat et al. 2015; Tabak et al. 2012). However, we were surprised to find relatively few empirical studies that identified systems level and organizational factors that affect EBI *scale-up* (Bradshaw and Pas 2011; Pas and Bradshaw 2012). In addition, much of the existing research is descriptive and has not tested if or how certain factors affect the scale-up of EBIs in public systems and the degree to which scaling up EBIs reduces population levels of behavioral health problems. Increasing EBI scale-up thus requires much more rigorous empirical research to create and test a comprehensive framework for scaling up EBIs, as well as evaluations to study the influence and impact on outcomes of the specific factors that impede and facilitate scale-up.

Given the dearth of prior research and literature, the list of factors affecting scale-up described in this paper should be viewed as preliminary and in need of further examination and testing. Nonetheless, the fact that we identified a common set of factors likely to affect EBI scale-up in all five public systems is noteworthy. A primary goal of this paper is to draw attention to these common factors and inspire prevention scientists, policy makers, and practitioners to identify and evaluate ways to address the barriers and promote the facilitators of EBI scale-up.

We consider a significant contribution of our work to be the delineation of how public systems operate, including their decision-making structures and funding streams. The paper provided a comprehensive overview of federal and some state and/or local government statutes, guidance, and funding mechanisms related to EBIs because these are necessary elements for ensuring EBI scale-up. Additional statutes requiring the use of EBIs, more long-term funding for EBIs, and greater support and oversight to ensure high-quality implementation are all needed to achieve population-level improvements in public health and well-being. We also recognize that private funds, as well as joint public/private ventures, can help promote EBI scale-up.

Although necessary, top-down edicts are not sufficient to change how systems and individuals within systems operate. Scaling up also requires that leaders at all levels learn more about and become more supportive of EBIs. They must engender support from staff and ensure that they are appropriately trained and supported in their efforts to deliver EBIs. It is also important to embed into systems user-friendly methods for collecting and analyzing data, create a culture that values and uses these data to improve services, and be able to link data within and across systems. There is also a public responsibility to promote EBI implementation, and we call on communities to become more actively involved in these efforts. In addition, purveyor and intermediary organizations can help build community capacity to select, implement, and sustain EBIs that address local needs and are responsive to the community. Authentic and sustained research-practice partnerships are also needed to support the scale-up of EBIs in ways that are culturally and contextually relevant.

These and other recommendations for scaling up EBIs are summarized in Table 2. Given the overlap in factors that existed across systems, these recommendations are not system-specific.^6^ In addition, we intentionally chose not to link particular recommendations with particular actors given that scaling up will require collaborative action between and across multiple actors and systems. To that end, the recommendations are grouped into three broad categories to denote actions to (1) increase public policies and funding to facilitate the creation, testing, and scaling up of EBIs; (2) inspire further research and evaluation of scale-up efforts; and (3) promote public support for EBIs, community capacity to implement EBIs at scale, and partnerships between community stakeholders, policy makers, practitioners, and scientists. We hope that the prevention science, practice, and policy communities will be inspired by the findings articulated in this paper and begin to co-create opportunities for EBI scale-up at a systems level, since EBIs will ultimately be adopted, implemented, and sustained within and/or at the direction of public systems.

**Table 2:** Recommendations for promoting EBI scale-up in US public systems

Public policy and funding	
1. Expand efforts to codify the use of EBIs in statutory language, with statutes written to define EBIs in ways that prioritize interventions tested in rigorous evaluations but also allow some flexibility in evaluation standards	
2. Ensure that statutes require EBIs and that the regulations and guidance related to these statutes include strong accountability procedures to enforce their requirements	
3. Increase the use of larger, more sustainable funding streams to support EBIs	
4. Increase the use of discretionary grants to promote innovation in EBI development, evaluation, and scale-up	
5. Support the creation and use of user-friendly data systems, that can be linked within and across systems, to monitor different types of EBIs, including their implementation and short- and long-term outcomes at county, state, and federal levels	
Research and evaluation	
6. Develop and test frameworks for scaling up EBIs that include the factors identified in this paper and measure the impact of EBI scale-up on population-level processes and outcomes	
7. Conduct case studies of specific EBIs to identify specific factors that influence scale-up and outcomes	
8. Investigate the capacity of scaled up EBIs to reduce disparities in behavioral health outcomes for disadvantaged sub-populations	
9. Conduct economic analyses of EBIs that are scaled up in public systems to determine financial costs and benefits	
10. Conduct research that investigates how to “optimize” EBIs to identify their core components with the goal of creating more efficient interventions with greater scale-up potential	
11. Examine how adaptations made to EBIs during the scaling up process, especially those made to accommodate system needs and resources, affect outcomes	
12. Assess the efficacy and effectiveness of purveyor and intermediary organizations to increase EBI scale-up	
13. Evaluate the types and extent of technical assistance required to scale-up EBIs and ensure they are well implemented	
14. Examine the process of disinvestment in and decommissioning of EBIs to determine how systems can make room for new EBIs	
Community support and partnerships	
15. Promote active partnerships between scientists, policy makers, practitioners, and community members, within and across systems	
16. Encourage policy makers, practitioners, and community members to identify the types of EBIs that need to be created and scaled up	
17. Promote the use of community-level coalitions with multi-sector representation to increase public support for EBIs	
18. Promote the capacity of communities to conduct needs assessments and select the best-fitting EBIs from “what works” registries	
19. Create more knowledgeable, supportive, and effective systems leaders and staff through workforce development and training	

## Footnotes

1 This paper conceptualizes behavioral health problems broadly to include social, physical, mental health, and academic problems. These problems may manifest, for example, as delinquency, violence, teen pregnancy, high-risk sexual behaviors, obesity, substance use/abuse, depression, anxiety, poor academic performance, and school drop-out.

2 The MAPS IV Task Force considered adding “community” as a system to recognize the important role that community-based non-profit agencies and networks play in financing, promoting, and delivering EBIs. However, we decided that communities are not a system per se, but rather are embedded in local, state and federal public systems. Furthermore, community agencies are too diverse in their scope, functions, and outcomes of interest to be neatly categorized as one system. Similarly, we debated whether or not “early childhood” should be an additional public system but decided not to designate it as such because early childhood EBIs are delivered in multiple public systems (e.g., child welfare, education, and public health).

3 As stated in Footnote 1, *behavioral health problems* are defined broadly in this paper and are not the sole purview of the *behavioral health* system. Although the behavioral health system is concerned with reducing an array of mental, emotional, and behavioral disorders (see Table 1), its efforts focus on mental health and substance use/abuse.

4 The federal government also provides *categorical* funds that are similar to block grants, but these are usually allocated for a more narrowly prescribed set of services. For example, Medicaid is a categorical grant that can be used for EBIs and is being used in Washington State to support the Triple P program with parents in primary care settings (Steverman and Shern 2014).

5 For comprehensive descriptions of evidence-based registries and discussion of their differences and limitations, see Burkhardt et al. (2015), Means et al. (2015), and Neuhoff et al. (2015).

6 Given that systems differ in their organizational structures, flexibility, and funding and statutory contexts, the specific ways in which systems can facilitate scale-up are likely to differ. As part of the MAPS IV initiative, the behavioral health, education, and public health workgroups drafted policy briefs that identify system-specific challenges to EBI scale-up and recommendations for addressing these factors. Links to these three briefs are available on the SPR website (https://www.preventionresearch.org/). We encourage readers of this manuscript to share the briefs with their colleagues, particularly those working within these public systems.
